# Pattern of use of radiotherapy for lung cancer: a descriptive study

**DOI:** 10.1186/1471-2407-14-697

**Published:** 2014-09-23

**Authors:** Isabel Tovar, Jose Expósito, Javier Jaén, Enrique Alonso, Miguel Martínez, Rosa Guerrero, Juan P Arrebola, Rosario Del Moral

**Affiliations:** Radiation Oncology Department, Virgen de las Nieves Universitary Hospital, Granada, Spain; Institute of Oncology, Cartuja, Sevilla, Spain; Radiation Oncology Department, Puerta del Mar Universitary Hospital, Cádiz, Spain; CIBER en Epidemiología y Salud Pública (CIBERESP), Granada, Spain

**Keywords:** Non-small cell lung cancer, Radiotherapy, Clinical practice patterns, Small cell lung cancer

## Abstract

**Background:**

Lung cancer remains one of the most prevalent forms of cancer. Radiotherapy, with or without other therapeutic modalities, is an effective treatment. Our objective was to report on the use of radiotherapy for lung cancer, its variability in our region, and to compare our results with the previous study done in 2004 (VARA-I) in our region and with other published data.

**Methods:**

We reviewed the clinical records and radiotherapy treatment sheets of all patients undergoing radiotherapy for lung cancer during 2007 in the 12 public hospitals in Andalusia, an autonomous region of Spain. Data were gathered on hospital, patient type and histological type, radiotherapy treatment characteristics, and tumor stage.

**Results:**

610 patients underwent initial radiotherapy. 37% of cases had stage III squamous cell lung cancer and were treated with radical therapy. 81% of patients with non-small and small cell lung cancer were treated with concomitant chemo-radiotherapy and the administered total dose was ≥ 60 Gy and ≥ 45 Gy respectively. The most common regimen for patients treated with palliative intent (44.6%) was 30 Gy. The total irradiation rate was 19.6% with significant differences among provinces (range, 8.5-25.6%; p < 0.001). These differences were significantly correlated with the geographical distribution of radiation oncologists (r = 0.78; p = 0.02). Our results were similar to other published data and previous study VARA-I.

**Conclusions:**

Our results shows no differences according to the other published data and data gathered in the study VARA-I. There is still wide variability in the application of radiotherapy for lung cancer in our setting that significantly correlates with the geographical distribution of radiation oncologists.

## Background

Lung cancer (LC) is a worldwide health problem [1]. In Spain, approximately 20 000 new cases are reported each year and 18 000 individuals die from this disease. LC is the first cause of cancer mortality in men and the third in women (after breast and colorectal carcinomas) [1]. The incidence in women is 6-fold lower than in men but is increasing, as in other Western countries [2, 3].

Non-small cell lung cancer (NSCLC) accounts for 80% of all LCs, and the tumor load (stage) at the time of diagnosis is a critical factor for its clinical management [4]. According to clinical evidence accumulated over the past decades, optimal outcomes are obtained if tumors are treated in early stages, when surgery is more feasible. When this is not possible, there is a strong consensus that a multidisciplinary approach is warranted [5]. Thus, clinical guidelines recommend the combination of chemotherapy (CT) and radiotherapy (RT) in different schedules for patients with tumor stage II, IIIA, and IIIB. CT is recommended for the majority of patients with stage IV LC, depending on their performance status, and RT is used for palliative treatment [6–8]. CT-RT is the standard treatment for small cell lung cancer (SCLC) patients with limited disease. In general, preventive whole brain RT is recommended after CT-RT. CT is the treatment of choice for SCLC patients with extensive disease, and RT is used in palliative treatments [6, 7].

RT has proven to be an effective treatment in LC, with or without other therapeutic modalities [1, 9]. However, several studies [10, 11] have shown wide variations in its management among hospitals, including differences in utilization rate and schedules in both NSCLC and SCLC patients. Variability in medical practice (VMP) can imply worse outcomes, greater morbidity, higher social costs and lower cost-effectiveness. For these reasons, there has been an increase in VMP studies, which usually attempt to explain any geographic variations in terms of the accessibility of human and technical resources. In many cases, however, differences in the type of professional practice may play a role in this variability [12, 13].

We previously conducted a study that focused on the RT utilization rate and the patterns of RT application in patients with breast, lung, gynaecology and head and neck cancer (Variability and Appropriateness of Radiotherapy in Andalusia [VARA] project I) in Andalusian public hospitals in 2004 [14]. This study attributed the inter-hospital variability in RT management schedules, doses, fractionations to the low treatment unit: inhabitant ratio (three per million inhabitants) (r = 0.823/p = 0.001) and number of radiation oncologists (r = 0.888/p < 0.001). In addition, we found the greatest variability in RT for LC (for example, in NSCLC the administered dose ≥60Gy ranged between 0% and 28,1% according to the hospital). A program launched in 2004 to improve regional RT resources led to ratio of 4.2 treatment units per million inhabitants by 2006. The objective of the present study was to describe the variations in LC management among regional cancer centers in Andalusia during 2007.

## Methods

A retrospective longitudinal study was conducted during 2007 in all of the 12 public hospitals that offered RT treatments in Andalusia, an autonomous region of Southern Spain with 8.4 million inhabitants. These centers are distributed among the eight provinces of the region, ensuring coverage of the whole population. Only 10% of the total care is delivered in private healthcare facilities in the region.

We reviewed the clinical records and treatment of all patients who received external beam RT as primary treatment (after the diagnosis, excluding patients who receive RT for relapse or progression of the disease after the first treatment) for LC of any histological type or stage with radical or palliative intent based on the treatment intent recorded in the charts. Data were obtained from the hospital discharge information system (Minimum Basic Data Set), hospital cancer registries, and clinical management computer systems linked to the RT equipment (Varis®, Lantis® and Impac® networks). Demographic information was obtained from the Spanish National Statistics Institute (http://www.ine.es) [15], and estimates of the incidence of cancer in the Andalusian population and its distribution by histological type and stage were extrapolated from data from the Population Cancer Registry of Granada [16] and Carlos III Institute of Health, Madrid [2]. Trained researchers supervised by the staff at each center obtained patient data from the clinical records and individual treatment records.

Study variables included characteristics of the hospital (province, megavoltage units, and professionals), patient (age, gender, histological type, performance status estimated with Eastern Cooperative Oncology Group (ECOG) scale or Karnofsky scale, weight loss, and co-morbidity), and treatment (medical indication: therapeutic intent, total doses, fractions, nodal irradiation, delay, days of treatment, planning with 2D or 3D, electron linear accelerator or cobalt 60 treatment, and adverse effects).

Statistical procedures. Descriptive outcomes are shown as means, medians, standard deviations and confidence intervals. The bivariate analysis was performed using chi-square test and Student t-test. SPSS version 12.0 (SPSS, Chicago IL) was used for statistical analyses. The significance level was set at p < 0.05 and all tests were two-tailed.

Ethical considerations. This was a retrospective study with no diagnostic or therapeutic implications. The research was approved by the Andalusian Ethics Committee for Clinical Trials.

## Results

### Patients

Out of the 3051 diagnosed cases of LC during the study period in the population of Andalusia, we collected data on the 610 patients who received RT as primary treatment for the disease. The majority of patients were male (91%), and the median age was 65 years (65 ± 10.4 years); 37% of cases had squamous cell carcinoma, 17% adenocarcinomas, 15% large-cell undifferentiated carcinoma, 12% NSCLCs of other histologies, and 19% SCLC.

Missing data were related to performance status (47%), co-morbidity (26%), weight loss (44%), and toxicity (77%). However, 44% of cases (268 patients) had a good performance (ECOG 0–1) and 60% showed a weight loss ≤ 10%.

The patients were staged according to TNM 6^th^ edition [17].

### Hospital and treatments

The distribution of the results by province is shown in Table 1.

**Table 1:** **Distribution by province and proportion of patients treated with radical (R) or palliative intent (P)**

Provinces	LC patients treated with RT	LC patients treated with RT (%)	Patients diagnosed with LC	RT rate (%) ^*^
1	21 (R 7 P 14)	3 (R 33 P 67)	244	9
2	54 (R 40 P 14)	9 (R 74 P 26)	458	12
3	70 (R 32 P 38)	11 (R 46 P 54)	305	23
4	87 (R 77 P 10)	14 (R 89 P 11)	336	26
5	48 (R 28 P 20)	8 (R 58 P 42)	183	26
6	48 (R 39 P 9)	8 (R 81 P 19)	244	20
7	132 (R 76 P 56)	22 (R 58 P 42)	580	23
8	150 (R 57 P 93)	25 (R 38 P 62)	701	21
Total	610 (R 356 P 254)	100% (R 58 P 42)	3051	20

*Statistically significant difference p < 0.001.

Out of the 610 patients in the study, 58% were treated with radical therapy (8% with adjuvant RT post-surgery) and 42% were treated with palliative therapy. The diagnosis was NSLCL in 494 patients (81%) and SCLC in 116 (19%); 62% of the NSCLC patients had stage III disease, and 71% of the SCLC patients had limited disease (Table 2).

**Table 2:** **Distribution by stage and histology**

NSCLC N = 494 (81%)				SCLC N = 116 (19%)			
Stage	N (%) radical	N (%) palliative	Total (%)	Stage	N (%) radical	N (%) palliative	Total (%)
**I**	17 (6.2)	4 (2)	21 (4.2)				
**II**	33 (12.1)	8 (3.7)	41 (8.3)	**Limited**	82 (71)	-	82 (71)
**III**	179(65.3)	125 (57)	304 (61.5)				
**IV**	45 (16.4)	83 (37.3)	128 (26)	**Extended**	-	34 (29)	34 (29)
**n = 610**	274 (55.4)	220 (44.6)	494 (100)		82 (71)	34 (29)	116 (100)

Computed tomography-based RT treatment planning was performed in 95.1% of cases, and linear accelerator treatment was applied in 70.7%.

Associated CT was received by over half of the patients (sequential CT by 30.3%, concomitant CT by 34.34%, and both by 4.4%). As shown in Table 3, the most common CT regimen was a platin (cisplatin or carboplatin) combined with taxol 24.5%, gemcitabine 14.2% or vinorelbine 13.2% or etoposide 28.3%. Etoposide was used in SCLC.

**Table 3:** **Associated chemotherapy and regimens**

Associated chemotherapy			Regimen		
	Patients (%)			Patients (%)	
	NSCLC	SCLC		NSCLC	SCLC
**No**	163 (33)	16 (14)	**CDDP + Taxol**	15 (3)	-
**Sequential**	153 (31)	35 (30)	**Carbo + Taxol**	153 (31)	-
**Concomitant**	153 (31)	59 (51)	**CDDP + GMZ**	84 (17)	2 (2)
**Both**	25 (5)	6 (5)	**CDDP + VNB**	84 (17)	2 (2)
**Total**	494 (100)	116 (100)	**CDDP + VP-16**	15 (3)	53 (46)
			**Carbo + VP-16**	15 (3)	52 (45)
			**Other**	128 (26)	7 (5)
			**Total**	494 (100)	116 (100)

CDDP = cisplatin; Carbo = carboplatin; VNB = vinorelbine; GMZ = gemcitabine; VP-16 = etoposide.

### Radical radiotherapy in NSCLC

Radical RT was applied to 274 NSCLC patients, whose characteristics (gender and age) were similar to those of the whole series. Most of them had squamous cell carcinoma with an advanced stages; 58% had an ECOG of 0–1, and 42% did not show a weight loss > 10%. A regimen of ≥60 Gy with standard fractionation (1.8-2 Gy per fraction) was administered to 74% of the patients. The irradiation field contained the mediastinal area in 81% of cases. The interval between the ordering and commencement of the treatment was <30 days in 49.7% of patients. CT associated with RT was the most common approach (sequential in 32.6%, concomitant in 41.7%, and both in 5.1% of cases). The CT schedule was a platin (cisplatin or carboplatin) with taxol, vinorelbine, or gemcitabine.

### Radical radiotherapy in SCLC

Radical RT was applied to 82 SCLC patients, whose characteristics (gender and age) were similar to those of the whole series. The majority of patients had limited stage with good performance status (ECOG 0–1 in 87%), although weight loss was more frequent (57%). 97% of patients received doses ≥45 Gy with standard fractionation RT; (only 9 patients underwent a hypofractionated schedule). RT treatment was delayed for >30 days in 67%, probably due to the CT treatment. All patients were treated with CT (sequential in 26%, and concomitant in 63%). All except four patients received cisplatin (or carboplatin) plus etoposide.

Table 4 summarizes the characteristics of radical RT for NSCLC and SCLC.

**Table 4:** **Characteristics of radical radiotherapy in NSCLC and SCLC patients**

Radical radiotherapy in NSCLC									
Histology (%)		Stage (%)		RT dose (%)		RT delay (%)		Associated CT (%)	
**SCC**	48.1	**I**	6.2	**<60 Gy**	26	**<30 days**	49.7	**No**	20.6
**ADC**	21.7	**II**	12.1	**≥60 Gy**	74	**≥30 days**	50.3	**Seq**	32.6
**LCUC**	17.5	**III**	65.3					**Conc**	41.7
**Others**	12.7	**IV**	16.4					**Both**	5.1
**Radical radiotherapy in SCLC**									
**RT dose (%)**			**RT delay (%)**			**Associated CT (%)**			
**<45 Gy**		3	**<30 days**		33	**No**		0	
**≥45 Gy**		97	**≥30 days**		67	**Sequential**		26	
						**Concomitant**		63	
						**Both**		11	

SCC: squamous cell carcinoma, ADC: adenocarcinoma, LCUC: Large-cell undifferentiated carcinoma. Seq: sequential, Conc: concomitant.

### Palliative radiotherapy (NSCLC and SCLC)

The majority of patients undergoing palliative RT were diagnosed with NSCLC (86.6%), mainly squamous cell carcinoma (39%); 44.8% were with advanced stages, 30% had an ECOG of 2–4, and 44% showed weight loss.

The most frequent RT schedule (in 44.6% of these patients) was 30 Gy (10 fractions × 3 Gy per fraction) (Table 5). CT was not received by almost 40% of these patients. The interval from the consultancy to palliative RT was <15 days in 40% of the patients.

**Table 5:** **RT schedules for LC treated with palliative intent**

Radiotherapy schedule	Total (%)
**Conventional fractionation (1.8-2 Gy)**	16.5%
**3 fractions x 400 cGy**	0.4%
**15 fractions x 300 cGy**	10.3%
**10 fractions x 300 cGy**	44.6%
**5 fractions x 400 cGy**	12.9%
**2 fractions x 850 cGy**	9.9%
**1 fraction x 800 cGy**	1.8%
**Hyperfractionation**	0.4%
**Other**	3.2%

### Comparative study

Statistically significant differences among provinces were found in the histology and stage of cancers and in RT intent, fractionation, and associated CT (p < 0.05) (Tables 1 and 6).

**Table 6:** **Statistically significant differences among provinces**

Province	Histology (%)					Stage (%)	
	SCC	ADC	LCUC	Others NSCLC	SCLC	Early (I-II)	Advanced (III-IV)
**1**	33.3	11.1	0	33.3	22.2	24.9	75.1
**2**	28.3	28.3	19.6	13	10.9	25	75
**3**	38.3	18.3	20	3.3	20	2	97.9
**4**	31.1	13.5	29.7	2.7	23	13	87
**5**	29.3	4.9	22	31.7	12.2	12.9	87.1
**6**	57.1	21.4	2.4	2.4	16.7	11.2	88.9
**7**	33.3	19.8	4.2	25	17.7	8.5	91.4
**8**	46.6	13.6	10.2	2.3	27.3	10.7	89.4
**Province**	**Fractionation (%)**			**Associated CT (%)**			
	**Conventional (1.8-2 Gy)**		**Other**	**No**	**Yes**		
**1**	50		50.1	47.1	52.9		
**2**	63.9		36.2	30.4	69.6		
**3**	45.9		54	15.5	84.4		
**4**	90.7		9.4	13.9	86.1		
**5**	54.8		45.3	56.1	43.9		
**6**	92.9		7.2	10.8	89.2		
**7**	46.9		52.9	47.9	52.2		
**8**	53.8		46.2	31.9	68.2		

SCC: squamous cell carcinoma, ADC: adenocarcinoma, LCUC: Large-cell undifferentiated carcinoma. NSCLC: non small cell lung cancer. SCLC: small cell lung cancer.

Statistically significant differences (p < 0.001, with the exception of stage with p = 0,036).

SCC: squamous cell carcinoma, ADC: adenocarcinoma, LCUC: Large-cell undifferentiated carcinoma. NSCLC: non small cell lung cancer. SCLC: small cell lung cancer.

**Figure 1:**
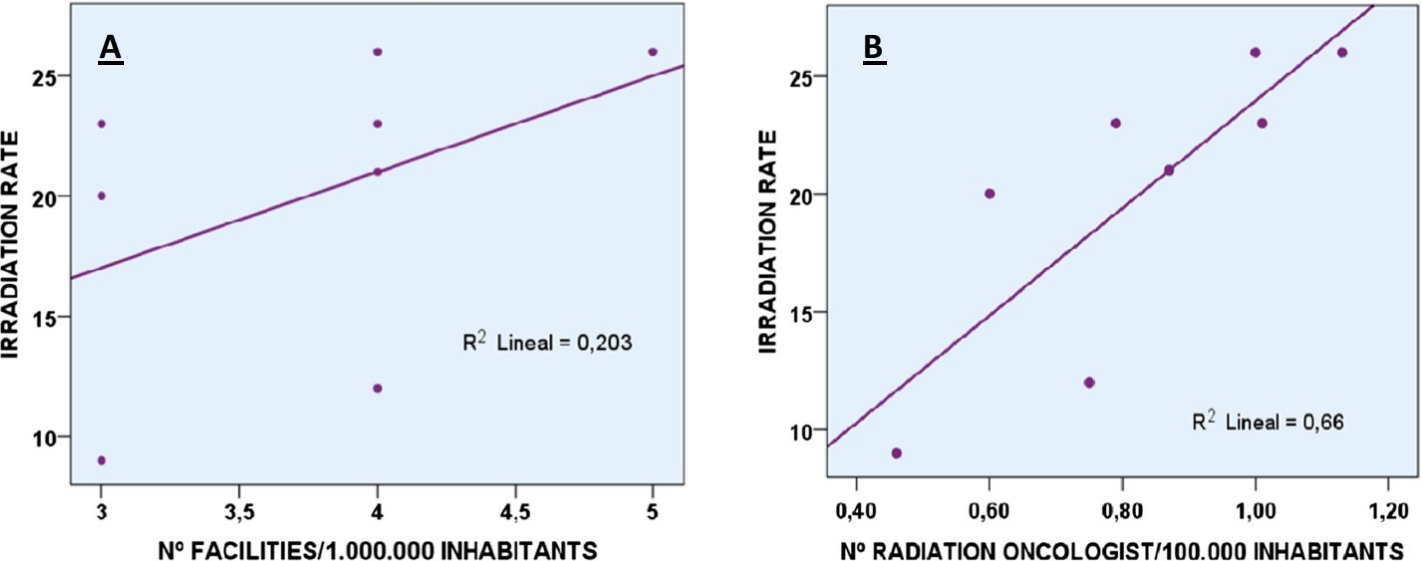
**Correlations with the radiation rate.** Correlation between irradiation rate and **(A)** number of radiotherapy treatment units (p > 0.05) and **(B)** number of radiation oncologists (p = 0.004).

Significant interprovincial differences in irradiation rate were found (p < 0.001) and were significantly correlated with the inter-provincial distribution of radiation oncologists (r = 0.66; p = 0.004) (Table 1, Figure 1).

We could not correlate the variability in the histology and stage of cancer with radiotherapy intent, fractionation, associated CT or radiation rate.

When we compared our data with those gathered from VARA-I study, we could observe an increase in total dose administered in both NSCLC and SCLC (p < 0.01), stage (only statistically significant in NSCLC, p > 0.01) with slight increase in advanced stages in our study, and patients treated with palliative intent (only statistically significant in NSCLC, p = 0.028). After the program to improve regional RT resources based on VARA-I results, the radiation rate increased by 4% from 16% in 2004 to 20% in 2007 (p < 0.001) (Table 7).

**Table 7:** **Comparison with data from VARA-I**

Dose (%)				Associated CT (%)			Stage (%)				Intent (%)			
		*VARA′04*	VARA *′*07		*VARA′04*	VARA *′*07			*VARA′04*	VARA′07			*VARA′04*	VARA´07
**NSCLC R**	**<60 Gy**	*147 (77.4)*	136 (27.5)	**No**	*106 (25.9)*	189 (31)	**NSCLC**	**I/II/III**	*332 (90)*	415 (84)	**NSCLC**	**R**	*212 (63.1)*	274 (55.4)
	**≥60 Gy** ^*^	*43 (22.6)*	358 (72.5)	**Yes** ^**^	*303 (74.1)*	421 (69)		**IV** ^#^	*37 (10)*	79 (16)		**P** ^##^	*124 (36.9)*	220 (44.6)
**SCLC R**	**<45 Gy**	*33 (54.1)*	1 (1.2)				**SCLC**	**LS**	*54 (79.1)*	82 (71)	**SCLC**	**R**	*54 (79.1)*	82 (71)
	**≥45 Gy** ^*^	*28 (45.9)*	115 (98.8)					**ES** ^**§**^	*14 (20.9)*	34 (29)		**P** ^**§**^	*14 (20.9)*	34 (29)
**Age (years)** ^**§**^				**Gender (%)**			**ECOG (%)**				**Radiation rate (%)** ^*^			
***VARA′04***		**VARA′07**			***VARA′04***	**VARA′07**		***VARA′04***		**VARA´07**	***VARA´04***		**VARA′07**	
*64,6 ± 10,7*		66 ± 10		♀	*33 (8)*	55 (9)	0 ^§§^	*94 (39.8)*		141 (43.9)	*416 (16)*		610 (20)	
				♂ ^^^	*383 (92)*	555 (91)	1	*85 (36)*		131 (40.7)				
							2	*36 (15.3)*		40 (12.5)				
							3	*18 (7.6)*		7 (2.2)				
							4	*3 (1.3)*		2 (0.7)				

NSCLC: non small cell lung cancer. SCLC: small cell lung cancer. LS: limited stage. ES: extended stage. R: radical. P: Palliative.

Differences: ^*^p < 0.01; ^**^p = 0.08; ^#^p = 0.011; ^##^p = 0.028; ^§^p > 0.1; ^§§^p = 0.02; ^^^p = 0.54.

Overall, our data were in agreement with other published data (Table 8) [18–25].

**Table 8:** **Comparison with other published data**

Study	Age	Gender (%)		Histology (%)				Stage (%)					
		♂	♀	SCC	ADC	LCUC	SCLC	I	II	III	IV	LS	ES
**Salmerón 2012** [18]	67	90	10	37	20	8	16	-	-	-	-	-	-
**Escuín 2009** [19]	70	-	-	38	-	-	20	-	-	-	-	-	-
**Prim 2010** [20]	67	93	7	39	19	10	20	10	9	34	47	31	69
**Escuín 2006** [21]	68	89	11	79 CPNCP			21	20	4	37	35	37	61
**Hernández 2004** [22]	68	85	15	38	17	12	30	-	-	-	-	-	-
**Santos-M 2005** [23]	67	89	11	33	30	4	13	21		35	42	44	55
**Estrada 2007** [24]	67	90	10	24	17	29	19	24	4	27	41	-	-
**Alonso-F 2005** [25]	67	92	8	58	29	5	19	27	7	35	31	47	53
**VARA-II**	**66**	**91**	**8**	**37**	**17**	**15**	**19**	**4**	**8**	**61**	**26**	**71**	**29**

SCC: squamous cell carcinoma, ADC: adenocarcinoma, LCUC: Large-cell undifferentiated carcinoma. SCLC: small cell lung cancer. LS: limited stage. ES: extended stage.

## Discussion

The role of RT as an effective treatment in LC is clearly established in clinical practice guidelines [6–8]. Most of the patients in this Spanish survey were male, as reported previously in this and other countries; there has been a progressive increase in LC incidence among women in Spain, but it remains considerably lower than in the USA [20, 26, 27].

The mean age at LC onset diagnosis is 65 years in our region, within the range of 63–67 years reported in other Spanish series, and the majority of LC patients are diagnosed with NSCLC in an advanced stage [20, 26, 27]. The most frequent histological type is squamous cell carcinoma, although the incidence rates for adenocarcinoma show a rising trend and may possibly become higher than those for squamous cell carcinoma in the future [20, 26, 27].

Thus, our results were not different from other published studies.

Majority (74%) of the NSCLC patients undergoing radical RT received ≥60 Gy with standard fractionation, and the irradiation field contained the mediastinal area in 81% of them. This is the standard dose recommended in the literature for this entity [6–8]. Why the remainder 26% of patients were treated with a total dose lower than 60 Gy is unknown. Concomitant RT-CT was applied in 41.7% of the patients with inoperable NSCLC, which can be considered a low percentage given the worldwide acceptance of concomitant RT-CT as standard treatment option in these cases [6–8, 28].

Almost all (97%) of the patients with SCLC who were treated with radical intent received doses ≥45 Gy, and 63% of them received concomitant RT-CT. Evidence has been published on the benefits of early RT administration in this situation [11]. Among various RT schedules used to treat LC with palliative intent, the most frequent was 300 cGy × 10 fractions. Differences in the schedules used are probably related to the different localizations of the sites under treatment.

Using a benchmark approach, Barbera L. et al. [29] estimated that initial RT was warranted in 49.3% of LC patients, and similar conclusions were reached by other authors using different study methods [30, 31]. The European ESTRO QUARTS project [32] recommended RT in up to 61% of LC patients. In the present survey, the irradiation rate was 20%, 4% higher than recorded in our region in 2004 (17% relative increase) but still low according to the estimated radiation rate, resulting in an underuse of RT for lung cancer [33].

We observed major and statistically significant variations among the eight provinces in the histology and stage of the disease and in RT intent, fractionation, associated CT (all p <0.05), and irradiation rate (p < 0.001).

The few studies that addressed VMP in the context of RT reported variations in medical practice and the underutilization of RT for LC [10, 11, 14, 34]. The initial articles by J E Wennberg on VMP [12, 13] systematized the possible causes of differences as follows:

1. Demand: clinical stage, histology, incidence, age, delay and distance from the hospital… couldn‘t explain this variability according to our results. Socio-economic differences were not analyzed in this study [35], however the Andalusian health system is a public system with universal free coverage. Patient support system is another important factor but, considering the public health system of Andalusia, we believe that the contribution of this factor would be minimal.
2. Service offer: accessibility. If epidemiological causes are ruled out, resource gaps or limitations may in part explain a low irradiation rate. In the present study, a lower irradiation rate was significantly correlated with a smaller number of radiation oncologists. In 2007, a mean of 4.2 megavoltage units were available per million inhabitants, and the treatment started a median of 41 days after it was ordered, with significant differences among hospitals.
3. Style of practice: medical practice patterns may explain the low irradiation rates found in our region [36, 37]. Most clinical trials have a control arm with standard treatment that does not include RT [38, 39].

In many cases, the variability detected in RT intent, fractionation, and associated CT may be attributable to differences in styles of professional practice. There is increasing evidence that unexplained variations in practice are widespread in oncology in general. However, the problem is not confined to oncology; it should be taken into account that RT programs maintain unusually good records, and the excellence of the information systems makes variations in practice highly visible [10].

Our results weren’t different from other published data and some differences (such as stage) can be due to our study only collecting patients treated with initial radiotherapy.

Regarding VARA-I, radiation rate, administered total dose, advanced stage and radiotherapy with palliative intent have increased. Thus, we can say that, although we have more confidence to give more total dose, the radiation rate has increased due to radiotherapy administered with palliative intent.

One study limitation is its retrospective and hospital population-based design, which means that the total number of patients irradiated for LC may be underestimated, because the patients who were treated in private centers and patients who might have travelled out of Andalusia weren’t collected. However, mobility inter-regions is an uncommon situation in our area, only 10% of the total care is delivered in private centers, and the data observed were consistent with the findings of the previous survey (VARA I) 3 years earlier and the descriptive analysis and the results obtained weren’t different from other published data. Other important limitation is the time between the year of the study (2007) and its publication (2013). Nowadays the health system is probably different regarding 2007. This is a common fact among the population study. However between VARA-I and VARA-II (period of time of three years) the situation was not very different. New techniques of treatment, as stereotactic body radiotherapy, are being implemented now, and no many centers have this technology. Therefore, our study can reflect very accurate picture of the use of RT in our health area.

## Conclusions

Although our results were in agreement with other published data and the irradiation rate has increased, this study shows some variability in care patterns for LC in our region. The irradiation rate significantly differs among the provinces and is correlated with the inter-provincial distribution of radiation oncologists. Significant inter-hospital differences were detected in the histology and stage of LC and in its management (RT intent, fractionation, and associated CT). According to the literature, variations in medical practice and underuse of RT are a worldwide problem that needs to be study and this research is an example of this. It is necessary to examine variations in practice and distinguish those that are appropriate from those that are not justified and must be eliminated.

## Abbreviations

LC: Lung Cancer
NSCLC: Non-Small Cell Lung Cancer
CT: Chemotherapy
RT: Radiotherapy
SCLC: Small Cell Lung Cancer
VMP: Variability in Medical Practice
VARA: Variability and Appropriateness of Radiotherapy in Andalusia
ECOG: Eastern Cooperative Oncology Group.
